# A 5‐year survival status prognosis of nonmetastatic cervical cancer patients through machine learning algorithms

**DOI:** 10.1002/cam4.5477

**Published:** 2022-12-08

**Authors:** Wenke Yu, Yanwei Lu, Huafeng Shou, Hong’en Xu, Lei Shi, Xiaolu Geng, Tao Song

**Affiliations:** ^1^ Department of Radiology Qingchun Hospital of Zhejiang Province Hangzhou Zhejiang China; ^2^ Cancer Center, Department of Radiation Oncology Zhejiang Provincial People's Hospital, Affiliated People's Hospital, Hangzhou Medical College Hangzhou Zhejiang China; ^3^ Department of Gynecology Zhejiang Provincial People's Hospital, Affiliated People's Hospital, Hangzhou Medical College Hangzhou Zhejiang China

**Keywords:** cervical cancer, machine learning, prediction, SEER, survival

## Abstract

**Background:**

Prediction models with high accuracy rates for nonmetastatic cervical cancer (CC) patients are limited. This study aimed to construct and compare predictive models on the basis of machine learning (ML) algorithms for predicting the 5‐year survival status of CC patients through using the Surveillance, Epidemiology, and End Results public database of the National Cancer Institute.

**Methods:**

The data registered from 2004 to 2016 were extracted and randomly divided into training and validation cohorts (8:2). The least absolute shrinkage and selection operator (LASSO) regression was employed to identify significant factors. Then, four predictive models were constructed, including logistic regression (LR), random forest (RF), support vector machine (SVM), and extreme gradient boosting (XGBoost). The predictive models were evaluated and compared using Receiver‐operating characteristics with areas under the curves (AUCs) and decision curve analysis (DCA), respectively.

**Results:**

A total of 13,802 patients were involved and classified into training (*N* = 11,041) and validation (*N* = 2761) cohorts. By using the LASSO regression method, seven factors were identified. In the training cohort, the XGBoost model showed the best performance (AUC = 0.8400) compared to the other three models (all *p* < 0.05 by Delong's test). In the validation cohort, the XGBoost model also demonstrated a superior prediction ability (AUC = 0.8365) than LR and SVM models (both *p* < 0.05 by Delong's test), although the difference was not statistically significant between the XGBoost and the RF models (*p* = 0.4251 by Delong's test). Based on the DCA results, the XGBoost model was also superior, and feature importance analysis indicated that the tumor stage was the most important variable among the seven factors.

**Conclusions:**

The XGBoost model proved to be an effective algorithm with better prediction abilities. This model is proposed to support better decision‐making for nonmetastatic CC patients in the future.

## Graphical abstract

In the training cohort, the extreme gradient boosting (XGBoost) prediction model showed the best performance as compared to the other three prediction models. In the validation cohort, the XGBoost model also demonstrated a superior prediction ability than the logistic regression model and the prediction model using support vector machine algorithm, with no statistical significance was observed between the XGBoost model and the prediction model using random forest algorithm.

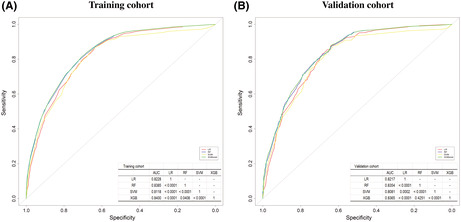

## 1 INTRODUCTION

Cancer of the cervix uterus (CC) continues to be a common malignant neoplasm and it ranks as the fourth major contributor to cancer‐related deaths among females on a global scale.^1^ The recent GLOBOCAN 2020 study estimated that there were exceeding 600,000 new cases of CC, with approximately 340,000 deaths estimated to occur in 2020.^2^ Curative surgical resection is the main treatment option for early stage CC, whereas definitive radio‐chemotherapy plays an alternative treatment option for locally advanced, nonmetastatic CC.^3^ With the application of human papillomavirus vaccines, cervical cytological screening, and other medical developments, the cumulative 5‐year overall survival (OS) rate for all stages is more than 60% among industrialized countries, while the corresponding figure is about 50% in many low‐/middle‐income countries.^4^ Therefore, it is urgent for clinics to learn how to predict the long‐term survival status of patients with nonmetastatic CC more accurately.

Traditionally, survival predictive models, like the logistic regression (LR) model, are usually based on proportional hazard regression models with different covariates depending on the study designs.^5^, ^6^ Since the beginning of this century, machine learning (ML) techniques have been used to create artificial intelligence models. These models have become more widely used in clinical settings.^7^ Among various ML algorithms, random forest (RF), support vector machine (SVM), and decision tree (DT) mainly are the three most classical algorithms that are popular.^8^, ^9^, ^10^ The RF algorithm is a kind of ensemble‐based machine learning method, based on a DT algorithm that can be used for classification. The result is the class selected by the most trees.^11^ The SVM algorithm employs a nonlinear mapping to transform the original data into higher‐dimensional data and searches for the optimal linear separating hyperplane within this new dimension. This algorithm finds the decision boundary using support vectors and margins.^8^, ^12^ Gradient tree boosting/gradient boosting decision tree (GBDT) is also known as a kind of DT and is further updated and improved as an extreme gradient boosting (XGBoost) algorithm by Chen and his coworkers in 2016.^13^ By adding regularization items to the cost function, the complexity of the model was controlled.^14^ This algorithm had proven its scalability and flexibility in a wide range of fields.^15^ In the application of ML algorithms for the prediction of CC, a cross‐sectional study has compared five different ML algorithms and found that the predictive model constructed on the basis of the DT algorithm could identify the most relevant predictors than other ML‐based classifiers.^10^ To our knowledge, similar applications of ML algorithms for the estimation of 5‐year survival status for CC patients have rarely been reported in the literature.

The National Cancer Institute's Surveillance, Epidemiology, and End Results (SEER) program was initially launched in 1975 and covers over one‐third of the overall US population currently.^16^ To reduce the cancer burden in the USA, it provides basic information on cancer statistics.^17^ Given that the SEER program is also an effective research tool, we aimed to evaluate and compare different algorithms for the prediction of survival status among patients with nonmetastatic CC in the current study. Hopefully, these findings will lead to better management of nonmetastatic CC in the future.

## 2 MATERIALS AND METHODS

### 2.1 Data collection and selection criteria

**FIGURE 1:**
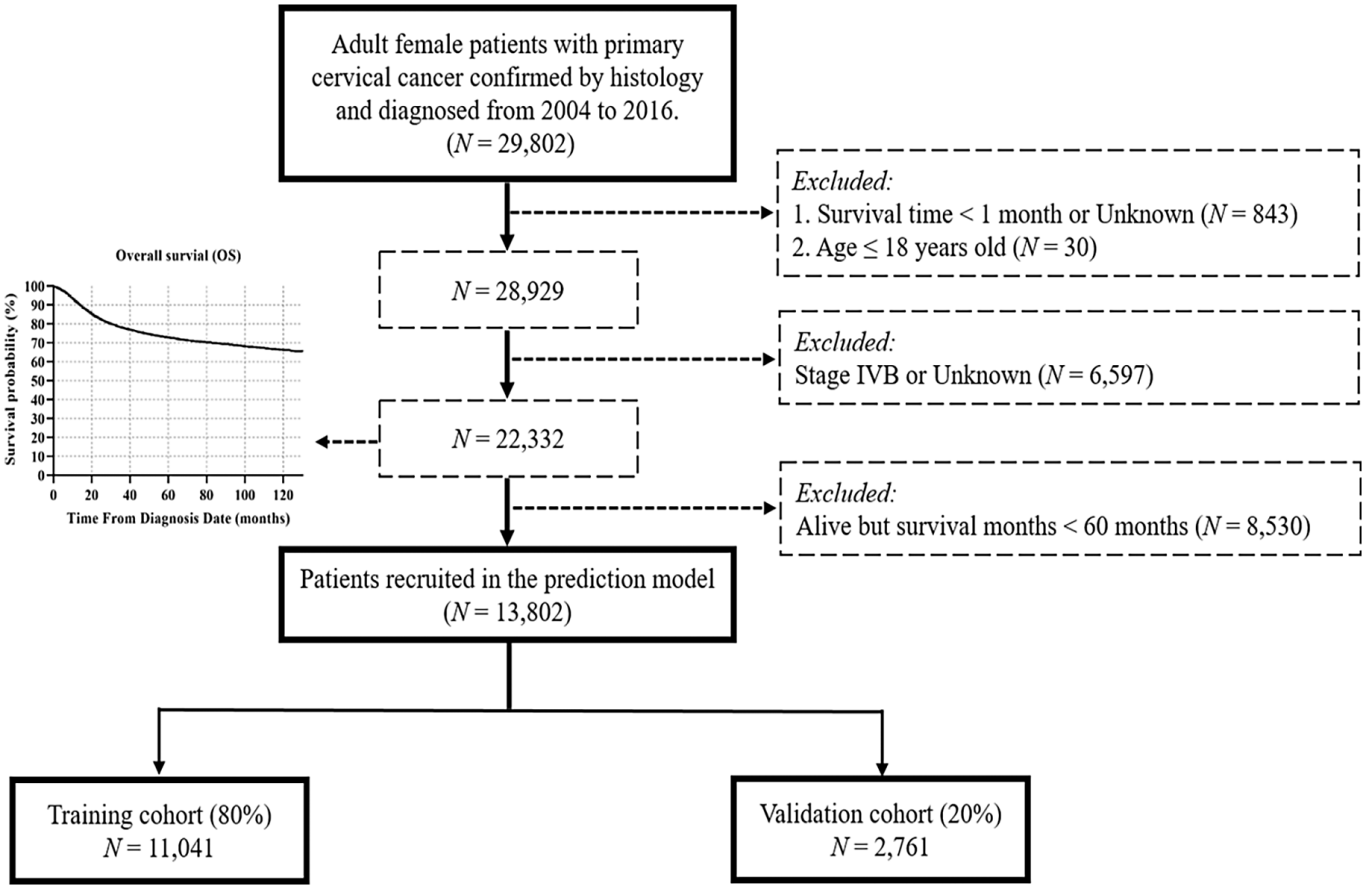
Illustration of the patient selection flowchart.

We used SEER*Stat version 8.3.9 software (accession number: 10248‐Nov2020) to retrieve patients' data that were further extracted based on the following criteria: (1) aged over 18 years old, (2) histopathologic diagnosis of CC between 2004 and 2016, and (3) primary stage of diagnosis (I‐IVA) of CC, with complete data about survival information, which incorporated survival months and status of survival (alive recorded as 0 or dead recorded as (1). The exclusion criteria were built based on the following parameters: (1) patients who had more than one primary cancer, (2) missing information on survival time (*N* = 10) or tumor stage (*N* = 2793), and 3) alive but survival time < 60 months at the last follow‐up mentioned in the database. The 5‐year survival status was set as the primary endpoint of the current study. The selection flow chart is illustrated in Figure 1.

### 2.2 Data preprocessing and selection

Variables for each nonmetastatic CC patient include age at diagnosis, marital status, race, histological type, differentiation grade, stage of the tumor, surgery at the primary site, regional lymph node surgery, use of RT and CT, size of the tumor, as well as the status of survival were retrieved and analyzed. The data extracted from the database were preprocessed to prepare them for modeling based on the classification methods applied in our previous studies.^18^, ^19^, ^20^ It should be pointed out that the latest 2018 International Federation of Gynecology and Obstetrics (FIGO) system of staging was abandoned because our previous study has demonstrated the nonsuperior prognostic impact of the 2018 FIGO system of staging over the 2014 FIGO system in patients diagnosed with locally advanced CC.^20^ Continuous data, such as age at diagnosis was established at 65‐years old as adopted by other SEER studies,^21^, ^22^ and tumor size was classified as a three categorical variable based on 40 mm (≤40 mm, >40 mm, and unknown).

### 2.3 Predictive models construction

The eligible data after the selection were randomly classified into the training and validation cohort according to the ratio of 8:2. As mentioned above, four predictive models were developed using the training cohort, including LR, RF, SVM, and XGBoost, with five‐fold cross‐validation, which was employed during the training of the ML algorithms. An easy‐to‐operate LR model is frequently applied to investigate the effect of trait variables on the target variable, which is typically a binary classifier, such as the survival status of CC patients at 5 years (alive or dead).^23^, ^24^ As its name implies, the RF model, which might reduce training variance and improve integration and generalization, is a machine learning classifier that adopts multiple trees to train and predict samples.^25^ SVM is another popular method in ML algorithms, and it is a kernel‐based algorithm that is used in the present study that transforms the feature space with multi‐dimensional attributes into two categories such as the survival status (alive or dead).^26^ For XGBoost, a start‐to‐art ML algorithm is characterized by Chen et al. in the aforementioned study.^13^

### 2.4 Models evaluations

To estimate the performance of each predictive model, the receiver‐operating characteristic (ROC) curves have been built and the areas under the ROC curves (AUCs) were identified to estimate the predictive performance of each predictive model and compared the outcomes of DeLong's test.^20^ Decision curve analyses (DCAs) were also employed to evaluate the usefulness of the four models constructed by previous steps for the prediction of 5‐year survival status and the methods for DCAs were also described in our previous study.^19^

### 2.5 Statistical analysis

Baseline characteristics of CC patients were described using descriptive statistics and frequency tables. We also compared the proportions of diverse tumor groups using the chi‐square (*χ* ^2^) test. The parameter of overall survival (OS) was calculated based on the time interval from the diagnosis of CC until death from any cause or the last follow‐up data, which is mentioned in the database. The survival curve was plotted using the Kaplan–Meier method. To balance between overfitting and underfitting among variables, least absolute shrinkage, and selection operator (LASSO) regression was used to determine the most crucial prognostic factors for predicting 5‐year survival status in the current study.

Statistical analysis was carried out using R software, version 3.6.2 (https://www.r‐project.org/), with the “caret”, “readr”, “Matrix”, “glmnet”, “pROC”, “rms”, “corrplot”, “randomForest”, “e1071”, “xgboost”, “rmda” and “nsROC” packages and SPSS 23.0 software (IBM Statistical Package for the Social Sciences). When the two‐sided *p* value of >0.05, it was found that there was no significant change in diagnostic performance across the predictive models.

## 3 RESULTS

### 3.1 Patient characteristics

In the SEER database, for the entire population of 22,332 patients without metastatic cancer, the estimated 5‐year OS rate was 72.84% (Figure 1). After taking into account the inclusion and exclusion criteria, 13,802 (61.8%, 13,802/22,332) CC patients who had accurate 5‐year survival status were enrolled in the final analysis. Table 1 presents the clinicopathologic characteristics and treatment modalities related to the training and validation cohorts. The chi‐square test revealed no statistically significant differences between the two cohorts (*p* > 0.05).

**TABLE 1:** Distribution of baseline characteristics of nonmetastatic cervical cancer patients from the SEER database

Characteristic	Training cohort (*n*, %)	Validation cohort (*n*, %)	*p* Value
Age at diagnosis(years)			
≤65	9385 (85.0)	2348 (85.0)	0.958
>65	1656 (15.0)	413 (15.0)	
Marital status			
Married	4677 (42.4)	1149 (41.6)	0.478
Unmarried and others	6364 (57.6)	1612 (58.4)	
Race			
White	8169 (74.0)	2040 (73.9)	0.989
Black	1675 (15.2)	419 (15.2)	
Others	1197 (10.8)	302 (10.9)	
Histology			
SCC	8663 (78.5)	2196 (79.5)	0.258
AC	1395 (12.6)	317 (11.5)	
Others	983 (8.9)	248 (9.0)	
Differentiation			
Well or moderately	4199 (38.0)	1029 (37.3)	0.448
Poorly or undifferentiated	3756 (34.0)	927 (33.6)	
Unknown	3086 (28.0)	805 (29.1)	
2014 FIGO stage			
I	6232 (56.4)	1504 (54.5)	0.265
II	2707 (24.5)	704 (25.5)	
III	1769 (16.0)	459 (16.6)	
IVA	333 (3.1)	94 (3.4)	
Surgery at the primary site			
No/Unknown	4592 (41.6)	1205 (43.6)	0.051
Local surgery	6449 (58.4)	1556 (56.4)	
Surgery of regional LN			
No/Unknown	6882 (62.3)	1750 (63.4)	0.307
Regional LN removed	4159 (37.7)	1011 (36.6)	
RT			
No/unknown	4504 (40.8)	1138 (41.2)	0.686
Yes	6537 (59.2)	1623 (58.8)	
CT			
No/unknown	5595 (50.7)	1383 (50.1)	0.583
Yes	5446 (49.3)	1378 (49.9)	
Tumor size (mm)			
≤40	3922 (35.5)	952 (34.5)	0.517
>40	3340 (30.3)	861 (31.2)	
Unknown	3779 (34.2)	948 (34.3)	

Abbreviations: AC, adenocarcinoma; CT, chemotherapy; FIGO, International Federation of Gynecology and Obstetrics; LN, lymph node; RT, radiotherapy; SCC, squamous cell carcinoma.

### 3.2 LASSO regression and variable selection

**FIGURE 2:**
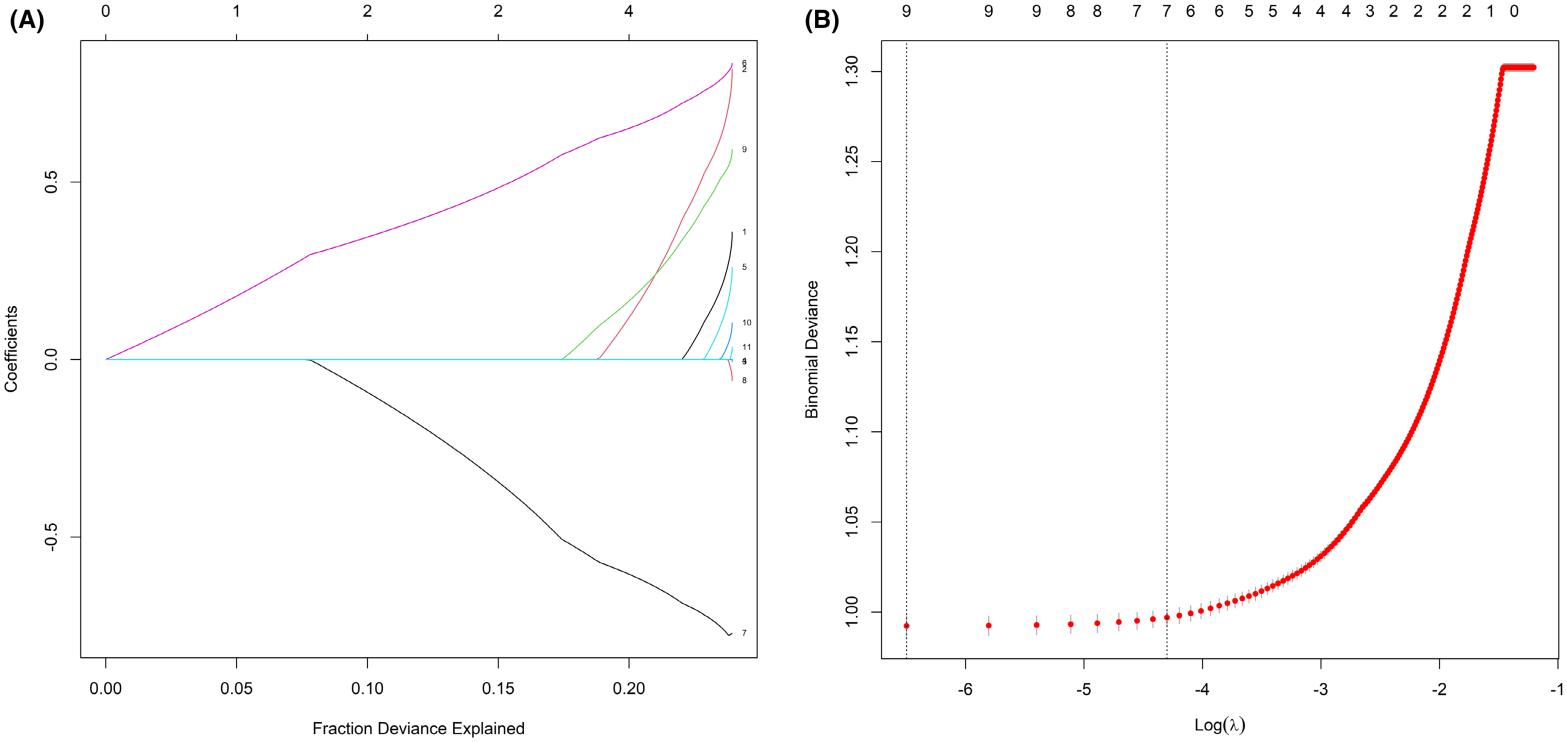
The results of the LASSO regression. LASSO, least absolute shrinkage and selection operator.

We have applied LASSO regression to reduce 11 characteristics of CC patients (nonmetastatic) in the training cohort. Initially, we excluded two variables (race [line 3] and tumor differentiation [line 4]) whose coefficients were zero in the regression model. Afterward, the remaining nine variables were entered into the LASSO regression model and were subsequently reduced to seven factors which are age at diagnosis (line 2, coefficient, 0.798), marital status (line 1, coefficient, 0.344), histology (line 5, coefficient, 0.244), 2014 FIGO stage (line 6, coefficient, 0.830), surgery at the primary site (line 7, coefficient, −0.771), RT (line 9, coefficient, 0.584), and CT (line 10, coefficient, 0.093) (Figure 2A). According to Figure 2B, the dashed vertical lines on the left represent the optimal value variables based on the minimum standard value.

### 3.3 Model evaluation

**FIGURE 3:**
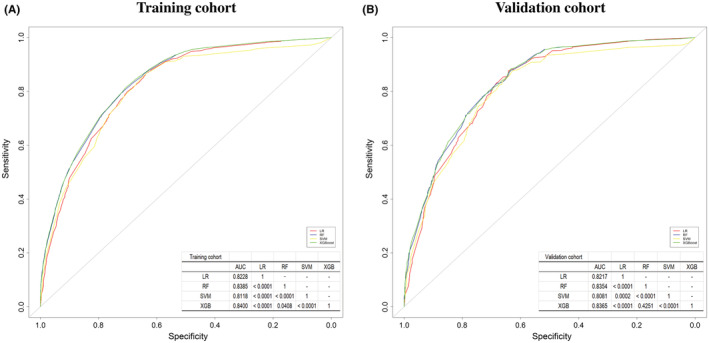
Receving‐operating characteristic curves and AUCs demonstrating the predictions of the four models: LR, RF, SVM, and XGBoost. (A) The training cohort and (B) the validation cohort. AUC, area under the curve; LR, logistic regression; RF, random forest; SVM, support vector machine, XGBoost, extreme gradient boosting.

To determine the accuracy of the four predictive models, ROC curves with corresponding AUCs were calculated for the training cohort (*N* = 11,041) and validation cohort (*N* = 2761). They have shown a roughly similar performance for the prediction of 5‐year survival status. Among these, the XGBoost model was observed to exhibit the best performance in the training cohort (AUC = 0.8400, 95% confidence interval [CI]: 0.8326–0.8474) compared with LR (AUC = 0.8228, 95% CI: 0.8150–0.8306), RF (AUC = 0.8385, 95% CI: 0.8311–0.8460), and SVM (AUC = 0.8118, 95% CI: 0.8034–0.8202). According to DeLong's tests, the XGBoost model showed statistically significant differences compared with the other three predictive models (all *p <* 0.05; Figure 3A).

In the validation cohort, the accuracy of the XGBoost model for predicting 5‐year survival status was superior (AUC = 0.8365, 95% CI: 0.8217–0.8513) than LR (AUC = 0.8217, 95% CI: 0.8063–0.8372), SVM (AUC = 0.8081, 95% CI: 0.7913–0.8249), and RF (AUC = 0.8354, 95% CI: 0.8205–0.8502), respectively. DeLong's tests have also shown that there were statistical differences between the XGBoost model and the other two predictive models (LR and SVM, both *p* < 0.01), where no statistical significance was observed between the XGBoost and RF model (*p* = 0.4251). (Figure 3B).

**FIGURE 4:**
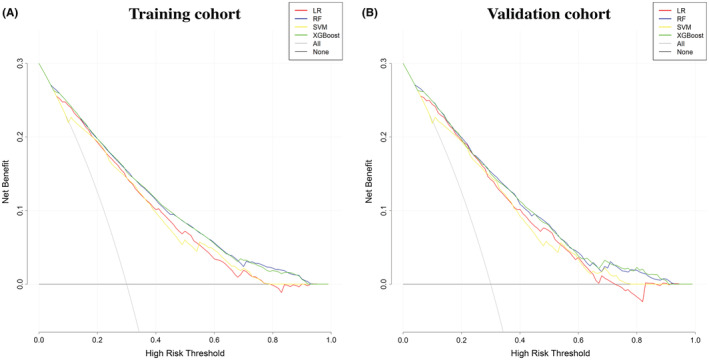
Decision curves showing the predictions of the four models: LR, RF, SVM, and XGBoost. (A) The training cohort and (B) the validation cohort. LR, logistic regression; RF, random forest; SVM, support vector machine, XGBoost, extreme gradient boosting.

In our study, DCAs of the four predictive models for the training and validation cohorts were also performed (Figure 4A, B). The decision curve of the XGBoost algorithm exhibited the greatest benefit over that of the other three models.

### 3.4 Feature importance of the XGBoost model

**FIGURE 5:**
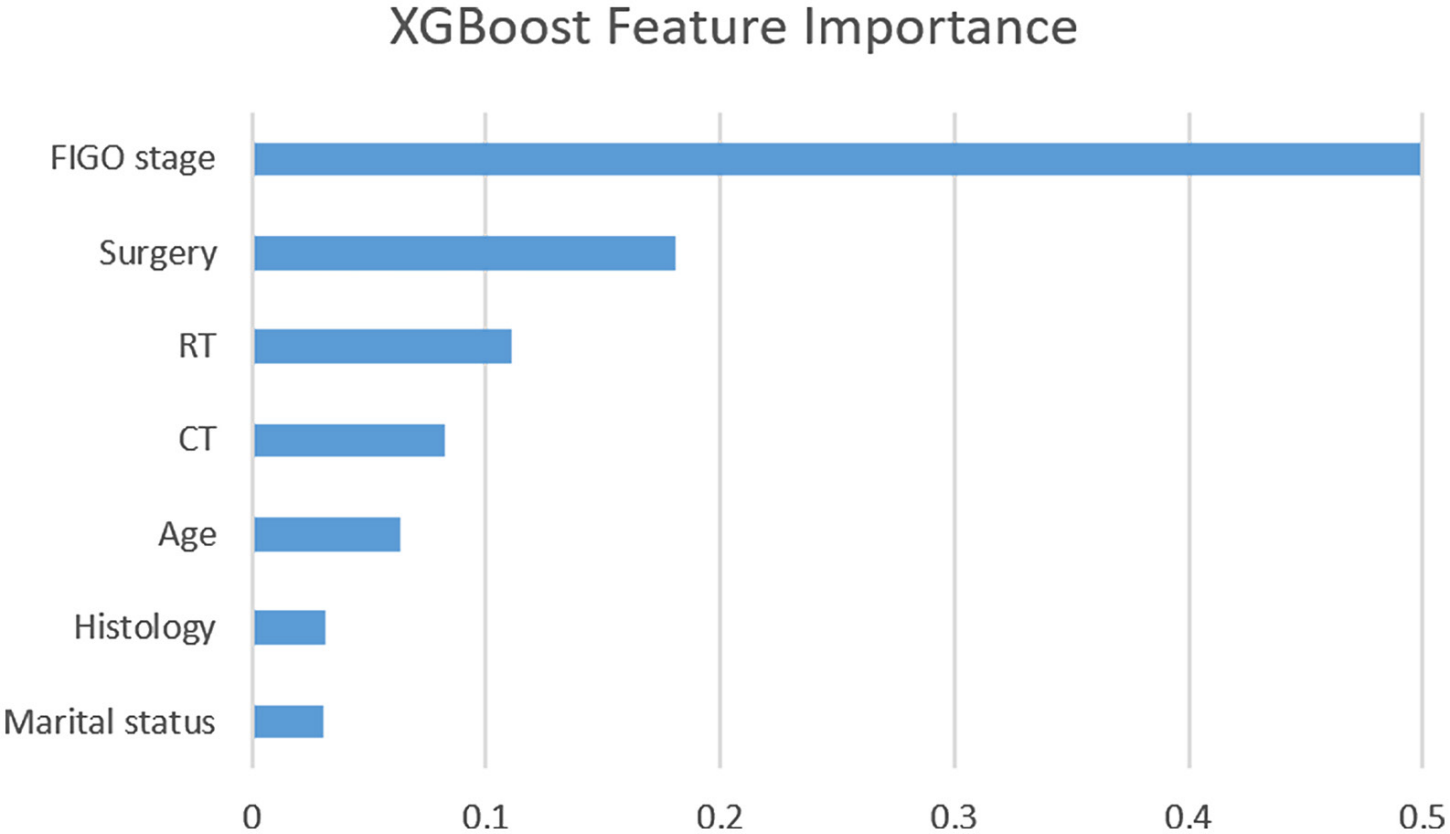
The XGBoost model was used to calculate the importance of seven features. XGBoost, extreme gradient boosting

The XGBoost predictive model demonstrated the best performance among the four predictive models in accessing the 5‐year survival status of nonmetastatic CC patients. Afterward, we analyzed feature importance within the selected seven factors in the LASSO regression model. The higher values indicated more importance for the prediction targets, including the tumor stage based on the 2014 FIGO staging system (corresponding importance rate, 49.93%), surgery at the primary site (18.07%), use of RT (11.23%), use of CT (8.28%), age at diagnosis (6.32%), histology (3.17%), and marital status (3.09%) (Figure 5).

## 4 DISCUSSION

In this study, we constructed four predictive models, including three models using ML algorithms for the prediction of the 5‐year survival status among nonmetastatic CC patients by combining seven routine clinical and treatment features selected by the LASSO regression model. Furthermore, although four predictive models performed in a nearly similar manner, Delong's comparison and DCAs revealed that the XGBoost algorithm had the best prediction ability compared with the other three models. Finally, the feature importance analysis of the XGBoost model indicated that the tumor stage based on the 2014 FIGO system of staging was the most important variable by combining big data with freely available demographic and clinicopathological characteristics in the SEER database.

The current clinic study did not set as a primary objective to describe in depth the differences between the algorithms within the four classifiers. In terms of the benefits of ML algorithms in cancer management, a similar application using ML algorithms as classifiers for the prediction of specific clinical endpoints was reported in recent years, which is mainly because some ML algorithms like XGBoost are gradually being added to clinical implementations. Wei and his colleagues evaluated four predictive models including LR, SVM, DT, and XGBoost for predicting lymph node metastasis (LNM) in prostate cancer using data gathered from the SEER database from 2010 to 2015. The XGBoost model showed the best predictive performance and the highest net benefit compared with the other three algorithms, with an AUC value of 0.883. The DCA plot also indicated that the XGBoost algorithm had the highest net benefit across the entire range of threshold probabilities compared with the other three predictive models.^27^ This algorithm has also been reported to be effective in predicting the prognosis of patients with esophageal cancer,^25^ non‐small‐cell lung cancers experiencing bone metastasis,^28^ and osteosarcoma.^29^ While the above studies used data from the SEER database, a multi‐institutional retrospective study was designed to construct and compare different ML models including LR, SVM, DT, RF, XGBoost, and LightGBM to predict survival in stage I–II CC patients who underwent complete resection in a real‐world setting. In total, 5112 patients with 22 baseline characteristics per patient were collected between 2006 and 2017. XGBoost algorithm displayed better prognostic predictive performance with an AUC of 0.906 for the study group that had all features enrolled in the model. Even after feature screening to reduce to a final seven variables, the XGBoost model still had a better performance of 0.808 compared with others.^30^ By reducing the bias‐related error and variance‐related error effectively, XGBoost algorithm avoids overfitting and makes the model best performing by repetitive operation among the four prediction models.^14^, ^29^ Similarly, a retrospective, pilot study compared the prediction accuracy of a deep learning neural network model to the classical Cox proportional hazard regression model in predicting the survival of 768 CC patients. There were 40 features in total for each patient. Comparing the deep learning model with the Cox regression model (mean absolute error, 29.3 vs. 316.2), the deep learning model performed better in terms of predictive accuracy. Further, the performance of the deep learning model could be enhanced if more features were incorporated than in the Cox regression model.^31^ Using these findings, the authors suggested that analytic models using modern statistical methods could provide clinicians with more accurate and meaningful survival information. Moreover, in the present study, by employing LASSO regression and comparing four different prediction models, our findings supported that ML algorithms demonstrated robust prediction ability for nonmetastatic CC patients and could be applied as support tools that could be integrated into daily clinical decision‐making.

On the other hand, the variable importance analysis of the XGBoost algorithm revealed that tumor stage was the most important feature for predicting 5‐year survival status among patients with CC. Based on our previous comparison of the prognostic impact of FIGO 2014 and 2018 staging systems for Stage IIB–IVA of CC, no significant difference in survival risk classification was observed for the updated 2018 FIGO staging system over the 2014 FIGO staging system.^20^ Another large sample study and a retrospective analysis conducted in the USA demonstrated that 53% of CC patients experienced stage migration where the most frequent tumor stage changes were observed from stages I–II to stage III and from stages II–III to stage IV. FIGO's 2018 staging system improved survival discrimination for stages I and IV patients, but great heterogeneity still existed among stage III patients based on the revised staging systems.^32^ Similar findings were also reported by other treatment centers.^33^, ^34^ Additionally, three treatment‐related variables of surgery, RT, and CT were following closely behind as indicated in Figure 5. Previously, a large cohort, retrospective analysis in Japan also demonstrated that for patients with localized diseases, CC patients who underwent surgery‐based treatment had a significant survival advantage over patients who had received RT,^35^ which was similar to the current findings.

The ML algorithms have been proven to provide more accurate disease classification and prediction of survival for CC patients, leading to a series of studies investigating how they can work with more clinical data such as radiomics^36^ in the literature. Kan et al. evaluated 970 radiomic features obtained from MRI sequences and seven clinical characteristics in 143 early stage CC patients for the prediction of LNM.^37^ The radiomic signatures demonstrated good discrimination between LNM and non‐LNM groups in both the training and validation cohorts. As for providing a postoperative treatment option for early stage CC patients with intermediate‐risk factors, Chu and his coworkers found that based on their risk classification model constructed by ML algorithms, the best AUCs for predicting 2‐ and 5‐year OS were 0.88 and 0.63, respectively. In addition, their study also indicated that the ML‐based prognostic model exhibits significant discrimination in predicting OS (*p* = 0.011). On the contrary, based on the traditional Sedlis criteria^38^ as the risk classification system, no significant difference in OS was observed within the cohort.^39^ Promising results incorporating ML algorithms with gene expression, molecular subtyping, and pharmacogenomics in CC patients were also reported recently.^40^, ^41^, ^42^

### 4.1 Limitation and future improvement

There are limitations to this study. First, data variability including transformations and normalizations is the major challenge for the widespread use of ML algorithms. Second, the SEER database identified only 11 features per patient, which would limit the predictive power of ML algorithms, not only for the XGBoost model. To build more accurate and effective predictive models in the future, we need to introduce more features for analysis. Third, theories of different ML algorithms are obscure and intricate. For clinicians to utilize these models efficiently, they need to be not only intelligible but also capable of estimating their uncertainties.^43^ Fourth, since this study was a retrospective cohort analysis, variables with clinical significance, such as the baseline characteristics of patients, including the status of human papillomavirus infection, hemoglobin levels, nutritional status, treatment‐related parameters, like surgery type, treatment duration, toxicities, and subsequent treatment modalities are not available in the SEER database and the results presented in the current study should be reconfirmed in external validation data sets in a real‐world setting. Finally, if the results generated in the SEER database can be applied to other populations, such as those in low‐ and middle‐income countries, needs to be confirmed in the futuristic studies.

## 5 CONCLUSIONS

In the current investigation, we first selected seven readily available factors including age at diagnosis, marital status, histology, tumor stage, surgery at the primary site, RT, and CT on the basis of the LASSO regression model. To evaluate the accuracy of the predictions for nonmetastatic CC patients' 5‐year survival status, four models were developed. Owning to the superiority of its inherent algorithm, XGBoost model was found to demonstrate satisfactory prediction accuracy according to AUC analyses and DCA curves in comparison with other algorithms. In addition, a variable importance analysis revealed that tumor stage based on the 2014 FIGO system of staging was the most significant feature in the XGBoost model. We propose that the developed ML algorithms like XGBoost could be constructed as an efficient clinical support tool for helping clinicians with the better management of nonmetastatic CC patients in the future.

## AUTHOR CONTRIBUTIONS

**Wenke Yu:** Conceptualization (equal); software (equal); writing – original draft (lead). **Yanwei Lu:** Data curation (supporting); formal analysis (supporting); methodology (equal); validation (equal); writing – original draft (supporting). **Huafeng Shou:** Data curation (supporting); formal analysis (supporting); methodology (supporting). **Hong'en Xu:** Methodology (equal); software (equal); validation (equal); visualization (equal). **Lei Shi:** Data curation (equal); methodology (supporting); software (supporting); validation (supporting). **Xiaolu Geng:** Formal analysis (supporting); methodology (supporting); software (supporting). **Tao Song:** Conceptualization (lead); formal analysis (lead); investigation (lead); visualization (equal); writing – review and editing (lead).

## CONFLICT OF INTEREST

The authors declare that they have no competing interests.

## ETHICAL APPROVAL

It was not necessary to get written informed consent for participating in the present research as the information contained in the SEER database has been de‐identified and is publically available following authorization. The present research was exempted from ethical assessment by the Institutional Review Board of Zhejiang Provincial People's Hospital. We hereby certify that the present research was conducted in conformity with the Declaration of Helsinki.

## Data Availability

The data sets produced for this work are accessible upon inquiry from the corresponding author.
